# Rapid Isolation of Functional *ex vivo* Human Skin Tissue-Resident Memory T Lymphocytes

**DOI:** 10.3389/fimmu.2021.624013

**Published:** 2021-03-22

**Authors:** Weijie Du, Daniel Lenz, Ralf Köhler, Erping Zhang, Carla Cendon, Jinchan Li, Mona Massoud, Joachim Wachtlin, Juliane Bodo, Anja E. Hauser, Andreas Radbruch, Jun Dong

**Affiliations:** ^1^Cell Biology, Deutsches Rheuma-Forschungszentrum Berlin, Institute of the Leibniz Association, Berlin, Germany; ^2^Central Lab for Microscopy, Deutsches Rheuma-Forschungszentrum Berlin, Institute of the Leibniz Association, Berlin, Germany; ^3^Sankt Gertrauden Krankenhaus, Berlin, Germany; ^4^Medizinische Hochschule Brandenburg, Neurrupin, Germany; ^5^Plastische und Ästhetische Chirurgie, Berlin, Germany; ^6^Immune Dynamics, Rheumatology and Clinical Immunology, Charité Universitätsmedizin Berlin, Berlin, Germany

**Keywords:** human skin, tissue-resident memory T cells, yield, epitope, collagenase IV, gentle tissue dissociation, cell isolation

## Abstract

Studies in animal models have shown that skin tissue-resident memory T (T_RM_) cells provide enhanced and immediate effector function at the site of infection. However, analyses of skin T_RM_ cells in humans have been hindered by the lack of an optimized isolation protocol. Here, we present a combinatorial strategy-the 6-h collagenase IV digestion and gentle tissue dissociation – for rapid and efficient isolation of skin T_RM_ cells with skin tissue-specific immune features. In comparison with paired blood circulating memory T cells, these *ex vivo* isolated skin T cells express typical T_RM_ cell markers and display higher polyfunctional properties. Moreover, these isolated cells can also be assessed for longer periods of time in *ex vivo* cultures. Thus, the optimized isolation protocol provides a valuable tool for further understanding of human skin T_RM_ cells, especially for direct comparison with peripheral blood T cells at the same sample collection time.

## Introduction

Recent research has provided compelling evidence that, in addition to circulating memory T cells, there are also significant non-circulating tissue-resident memory T (T_RM_) cells residing in many tissues, such as in the skin, lungs, gut, liver (1–5), and bone marrow (6–10). Most but not all these T_RM_ cells express CD69 (7, 11–13), which probably contributes to their retention in tissues (14–16). Similarly, most T_RM_ cells do not express the chemokine receptor CCR7 (3, 7). Animal models showed that skin T_RM_ cells mediate first lines of defense against previously encountered pathogens (1, 2, 17, 18). Approximately 2 × 10e10 resident T cells have been estimated to be present in normal human skin. This number doubles that of circulating T cells in the peripheral blood (19). However, present understanding of human skin T_RM_ cells has been challenged by the lack of an optimized isolation protocol. In this regard, various approaches have been utilized to isolate skin T_RM_ cells, such as EDTA isolation (19), collagenase P (20), collagenase IV digestion (19, 21), and skin explants (19). Nevertheless, these methods either suffer from low yield or require long-term *in vitro* culture periods.

To establish an optimized protocol for rapid and efficient isolation of skin T_RM_ cells, we have evaluated six different protocols in terms of the preservation of epitopes of interest, cell viability, and yield. Among these six approaches, the modified collagenase IV (M.CoIV) protocol, i.e., the combination of 6-h collagenase IV digestion and gentle tissue dissociation, outperformed other protocols and resulted in the highest viable cell number while robustly preserving critical surface marker expressions (such as CD4, CD8, and CD69). Importantly, the M.CoIV isolation procedure does not induce skin TRM cell activation and proliferation. Cytokine profiles of isolated skin memory T cells stimulated by SEB and anti-CD3/CD28 revealed functional capacities, to which the successfully isolated various types of antigen-presenting cells (APCs), such as dermal dendritic cells (DDCs) and Langerhans cells (LCs), may contribute.

## Results

### Characterization of Human Skin T Cells *in situ*

**Figure 1:**
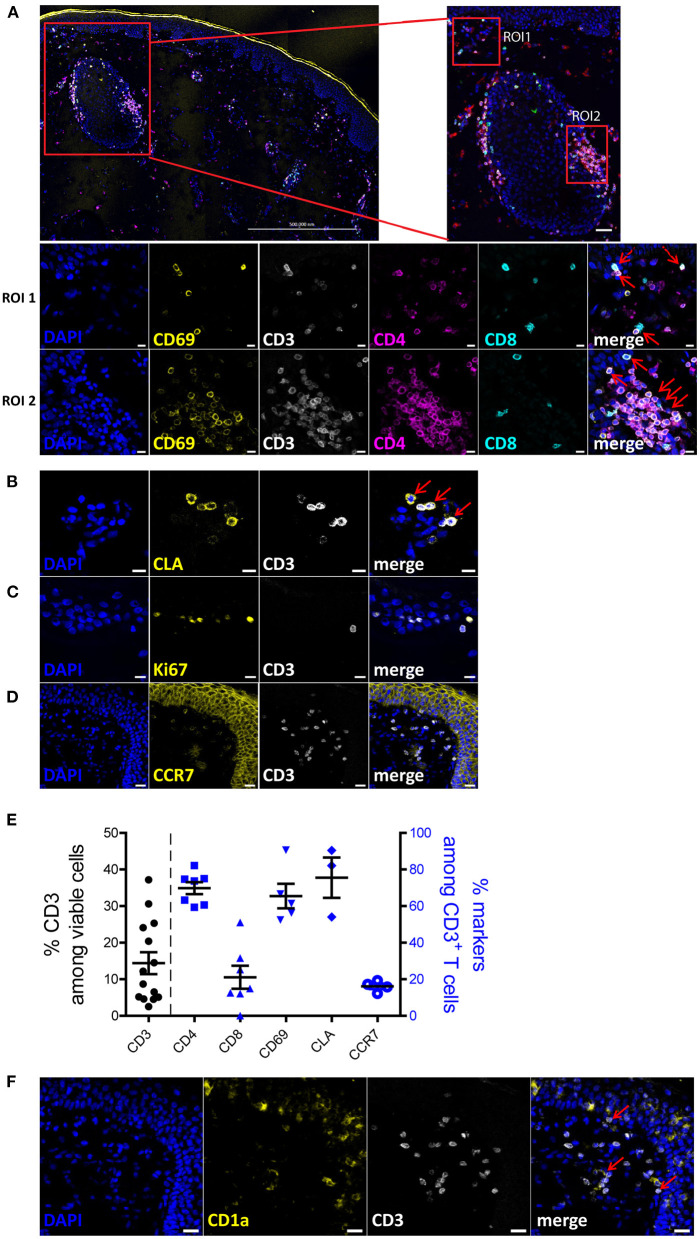
Phenotypic characterization of human skin cells *in situ*. **(A)** A 3 × 4 tile scan image of skin section stained with DAPI (blue), CD69 (yellow), CD3 (white), CD4 (violet) and CD8 (turquoise). Region of interest (ROI) 1 is a representative image of the cells located in the epidermis and ROI2 is a representative image of the cells around a hair follicle in the dermis. (**B–D,F**) Skin sections were stained with DAPI (blue) and CD3 (white) as well as one of the following: CLA **(B)**, Ki-67 **(C)**, CCR7 **(D)** or CD1a **(F)**. Scale bar: 500 μm for **(A)** upper left, 20 μm for **(A)** upper right, **(D,F)**; 10 μm for A-ROI1, A-ROI2, **(B,C)**. Co-expression of CD3^+^CD4^+^/CD8^+^ and CD69^+^ cells, and CLA^+^ and CD3^+^ cells are indicated by red arrows. Representative image sets from three independent experiments are shown. Scale bar: 20 μm for **(A,D,F)**; 10 μm for **(A–C)**. **(E)** Frequencies of CD3^+^ T cells among total cells (left y-axis) and frequencies of indicated subpopulations of T cells among CD3^+^ T cells (right y-axis), according to image cell quantification (*n* = 3; 14 fields).

To characterize the human skin T cells *in situ*, we performed immunofluorescence histology on 6 μm sections of eyelid and abdominal skin samples from healthy donors (Supplementary Table 1). Sections without antibody staining (Supplementary Figure 1A) or only with secondary antibody staining (Supplementary Figure 1B) were used as background controls. As shown in a large tile scan and the regions of interest (ROI) 1 and 2 in Figure 1A, CD8^+^ T cells localized in both the epidermis and dermis layers, while CD4^+^ T cells were mainly detected in the dermis and clustered around the hair follicles, with only few CD4^+^ T cells detected in the epidermis. Most CD3^+^ T cells, (CD4^+^ and CD8^+^), expressed CD69, indicating a tissue residency status of these T cells (Figure 1A). Skin CD3^+^ T cells expressed the skin homing markers, such as CLA (cutaneous lymphocyte-associated antigen) (Figure 1B) and did not express the proliferation marker Ki-67 (Figure 1C) or lymph node homing markers, such as CCR7 (Figure 1D). Quantitative analysis of immune cells present in the skin sections (Supplementary Figure 2) showed that, 14.4% (± 10.8) of skin cells were CD3^+^ T cells and among them 68.97% (± 8.06) and 24.56% (± 13.81) were CD4^+^ and CD8^+^ T cells, respectively, resulting in the ratio of CD4^+^ to CD8^+^ T cells of ~3:1 (Figure 1E). Additionally, while more than 65% of CD3^+^ T cells co-expressed CD69 and 75% co-expressed CLA, there were only 16% of CD3^+^ T cells co-expressing CCR7 (Figure 1E). The variation in frequencies especially of CD3^+^ T cells may reflect their uneven distribution in the skin. To identify the spatial distribution between T cells and dendritic cells, CD1a was concomitantly used with CD3 in the immunofluorescence staining. We observed that CD1a^+^ dendritic cells mainly resided in the epidermis layer and were close to CD3^+^ T cells (Figure 1F). Similarly, T cells expressing CD69 were also identified in the dermis of abdominal skin samples (Supplementary Figure 3A), although a strong autofluorescence signal in the FITC channel was detected (Supplementary Figure 3B), likely due to the intensive collagen fiber structures present in the abdominal skin. Together, these results suggest that normal human skin T cells are resting and qualify as T_RM_ cells.

### The Modified Collagenase IV Protocol Best Preserves Cell Surface Markers of Interest With High Cell Viability and Yield

**Figure 2:**
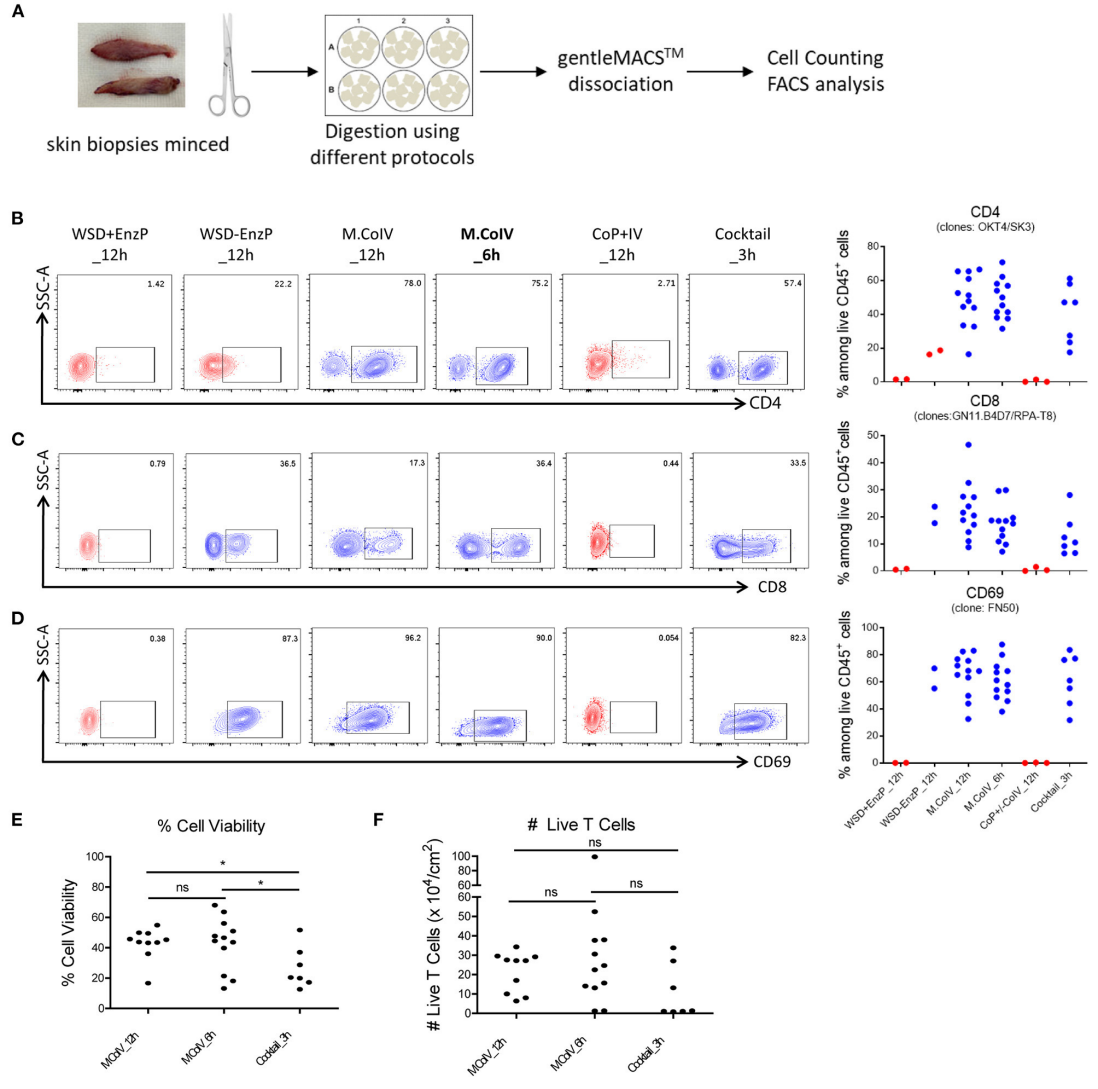
Modified Collagenase IV protocol best preserves the epitopes of surface antigens with high cell viability and yield. **(A)** Schematic workflow of isolating cells from human skin samples. **(B–D)** Frequencies of CD4^+^ **(B)**, CD8^+^ **(C)** and CD69^+^ **(D)** T cells among live CD45^+^ lymphocytes isolated by six different isolation protocols: (1) WSD+EnzP_12 h (*n* = 2), (2) WSD-EnzP_12 h (*n* = 2), (3) M.CoIV_12 h (*n* = 12), (4) M.CoIV_6 h (*n* = 12), (5) CoP+/-CoIV_12 h (*n* = 3), and (6) Cocktail_3 h (*n* = 7). Each dot represents data obtained from one donor. Red dots showing cells isolated from skin samples using protocols that did not preserve the CD4 epitope. **(E)** Frequencies of viable cells and **(F)** the total number of viable T cells isolated by using the M.CoIV_12 h, M.CoIV_6 h and cocktail_3 h isolation protocols. Statistical significance was calculated by two-tailed, unpaired *t*-test with Welch's correction. *p* < 0.05 (^*^).

To optimize the protocol for isolating human skin T cells, skin samples were minced and subjected to six reasonable protocols, each including a 3-, 6-, or 12-h enzymatic digestion (Figure 2A). These protocols are: combination of 1) a 12-hour collagenase IV digestion, i.e. modified collagenase IV digestion (M.CoIV)_12h; 2) M.CoIV_6h; 3) whole skin dissociation plus enzyme P digestion (WSD+EnzP_12h); 4) WSD-EnzP_12h (without enzyme P digestion); 5) CoP+CoIV_12h; or 6) cocktail of enzymes (collagenase I, elastase, hyaluronidase, and trypsin inhibitor) (Cocktail_3h), with gentle tissue dissociation (Supplementary Table 2). Cell isolated using these protocols were compared for expressions of CD45, CD3, CD4, CD8, CD69, CLA, and CCR7 among viable cells by flow cytometry. Notably, the modified collagenase IV (either 6- and 12-h digestion time) and cocktail protocols were the best to preserve the epitopes of antigens, such as CD4 (Figure 2B), CD8 (Figure 2C), and CD69 (Figure 2D). In terms of cell viability, significantly higher percentages of viable cells were isolated when using the M.CoIV_12 h and M.CoIV_6 h protocols (42.30 ± 5.01% and 42.36% ± 3.31%, respectively) than the cocktail_3 h protocol (26.33 ± 5.14%) (Figure 2E). In terms of viable T cell number, the M.CoIV_6 h protocol isolates more cells (28.73 ± 7.68 × 10^4^ live T cells per cm^2^) than the M.CoIV_12 h and cocktail_3 h protocols (19.29 ± 3.25 × 10^4^ and 10.81 ± 5.29 × 10^4^ live T cells per cm^2^, respectively) (Figure 2F). Thus, the M.CoIV_6 h protocol significantly outperformed other isolation protocols, representing an optimized protocol for isolating skin T cells.

### Characterization of *ex vivo* Skin T Cells

**Figure 3:**
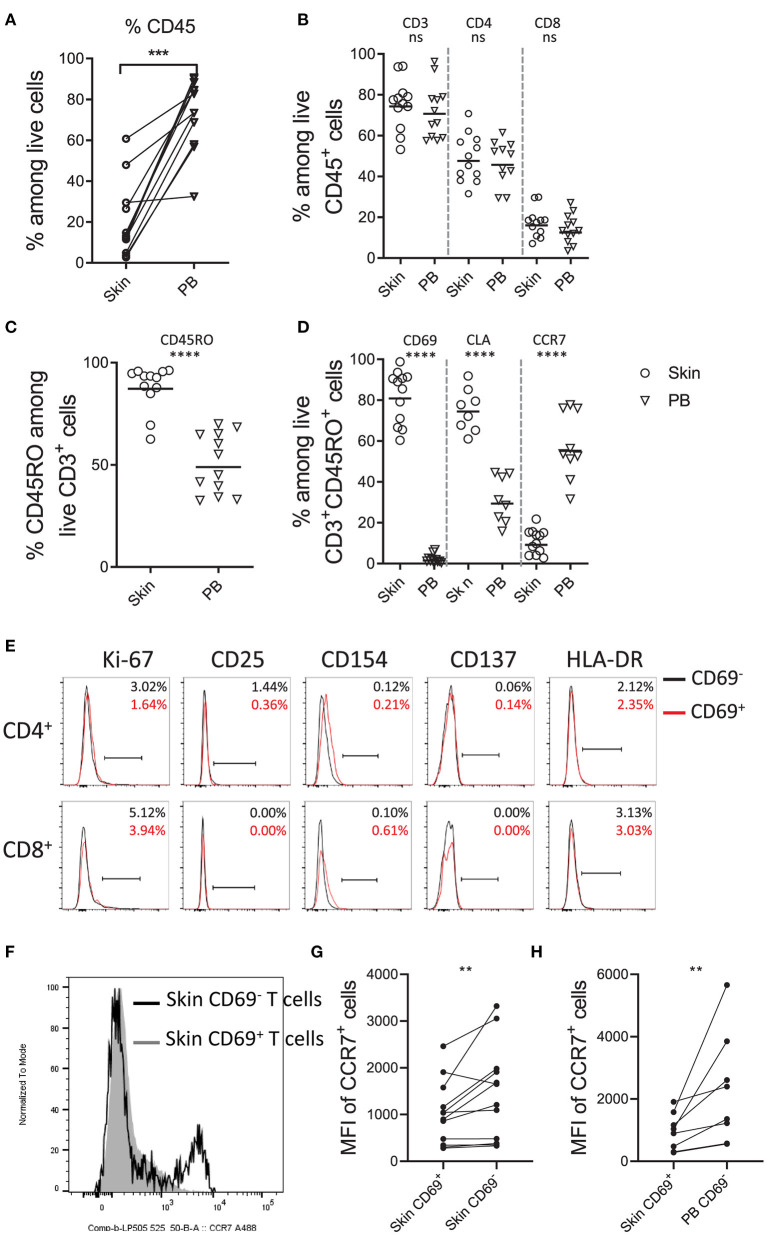
Phenotypic characterization of skin T cells by flow cytometry. Frequencies of CD45^+^ lymphocytes **(A)**, CD3^+^, CD4^+^, and CD8^+^ T cells **(B)**, CD45RO^+^ memory T cells **(C)**, and CD69^+^, CLA^+^ and CCR7^+^ cells among memory CD3^+^ T lymphocytes **(D)** in paired **(A)** and unpaired human skin and peripheral blood samples **(B–D)**. **(E)** Overlay of histograms showing the percentages of cells expressing proliferating and putative activation markers (Ki-67, CD25, CD154, CD137 and HLA-DR) on CD69^−^ (black line) and CD69^+^ (red line) memory CD4^+^ and CD8^+^ T cells. Percentages are shown in the upper right of each plot. Representative data from more than 10 independent experiments are shown. **(F)** Overlay of histograms showing the expression of CCR7 on CD69^+^ (filled gray area) and CD69^−^ (black line) skin T cells. Comparison of MFI (Mean Fluorescence Intensity) of CCR7^+^ cells between skin CD69^+^ and CD69^−^ T cells **(G)** and between skin CD69^+^ and blood CD69^−^ T cells. In **(A,G,H)**, Wilcoxon matched-pairs signed rank test, two-tailed; in **(B–D)**, unpaired *T*-test with Welch's correction, two-tailed. ^****^P < 0.0001, ^***^P < 0.001, ^**^P < 0.005, ns.

Using the optimized isolation protocol M.CoIV_6 h, we next characterized cells isolated from 12 (including 8 paired) skin samples in comparison with peripheral blood samples of 50- to 80-year-old individuals by flow cytometry (Supplementary Figure 4). Compared to blood, skin contained significantly lower frequencies of CD45 expressing lymphocytes (72 vs. 20%) (Figure 3A). However, among CD45^+^ lymphocytes, frequencies of CD3^+^ T cells as well as CD4^+^ and CD8^+^ T cells were comparable between skin and blood, resulting in the ratio of CD4^+^ to CD8^+^ T cells of 3:1 (Figure 3B), in line with that of skin T cells *in situ* (Figure 1E). The majority of skin T cells expressed CD45RO (87.93%), indicating a memory phenotype, whereas only approximately half of blood T cells (50.90%) expressed CD45RO (Figure 3C). Moreover, *ex vivo* skin memory T cells expressed the tissue resident markers such as CD69 (81.86%) and skin homing molecule CLA (75.08%) but rarely tissue egress markers, such as CCR7 (10.91%), in contrast to blood T cells (57.37%) (Figure 3D). Among CD3^+^ T cells, except for CCR7 (16 vs. 10%), the frequencies of these markers by *ex vivo* skin CD3^+^ T cells were similar to those by *in situ* skin CD3^+^ T cells (Figure 1E), suggesting that the M.CoIV_6 h protocol enables isolation of proportional skin cells.

Studies have shown that steady-state CD69^+^ T_RM_ cells from other tissues, such as the bone marrow (7), are resting in terms of activation. To test whether that would be also the case for normal skin T cells, we analyzed the expressions of proliferation marker Ki-67 and putative activation markers CD25, CD154, CD137 and HLA-DR on *ex vivo* skin T cell subsets isolated using the M.CoIV_6 h isolation protocol. Similar to CD69^−^ memory CD4^+^ and CD8^+^ T cells, CD69^+^ memory CD4^+^ and CD8^+^ T cells did not express these analyzed proliferation or activation markers (Figure 3E). This was not due to downregulation of these markers that might be potentially induced by the isolation procedure, in control experiments where T cells expressing these markers there was no downregulation of their expression following the isolation procedure (Supplementary Figures 5A,B). In agreement with T_RM_ features described from other tissues (3, 7), CD69^+^ skin T cells significantly downregulated CCR7 both in frequency (Figure 3F) and expression levels (Figures 3G,H), in comparison with their CD69^−^ counterparts in the skin (Figures 3F,G) or paired blood (Figure 3H). Together, these results describe a steady-state, memory T cell population as resident in the normal human adult skin. Furthermore, they demonstrate that the optimized M.CoIV_6 h isolation protocol does not activate skin T cells.

### Various Types of Antigen-Presenting Cells Can Be Isolated From the Human Skin by the M.CoIV Protocol

**Figure 4:**
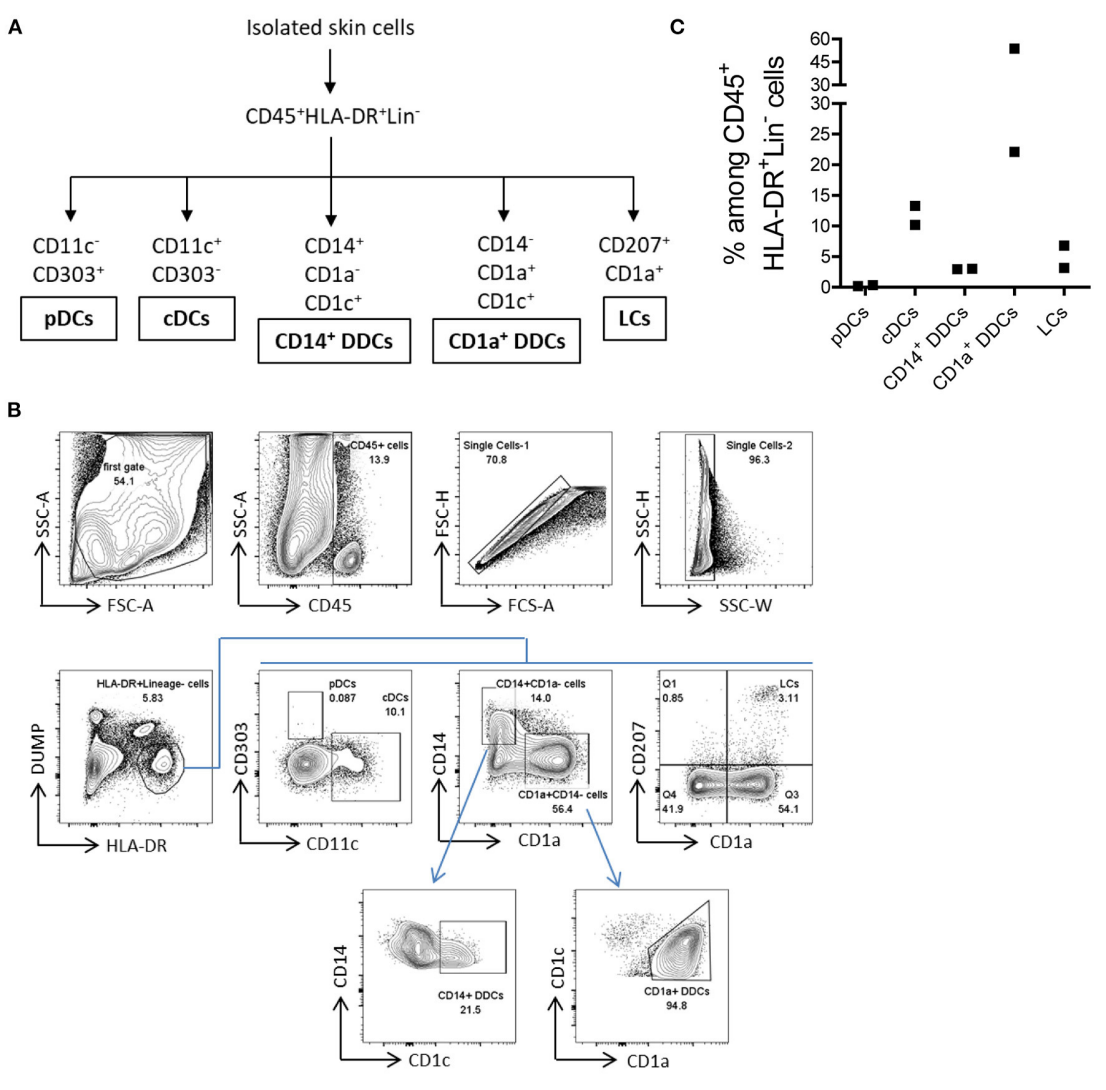
Isolation of five major types of APCs from normal human skin by using the M. CoIV_6 h protocol. **(A)** Classification of five main types of APCs distinguished by their surface markers (22, 23). **(B)** Gating Strategy for analyzing APCs from *ex vivo* human skin cells. Lineage markers included CD3, CD20, CD34, and CD56. Dead cells were excluded by DAPI staining. pDCs and CDCs were distinguished by CD11c against CD303. CD14^+^ DDCs and CD1a^+^ DDCs were distinguished by CD1a against CD14 and further based on the CD1C expression. LCs cells were gated based on the expression of CD1a and CD207. **(C)** Frequencies of *ex vivo* pDCs, cDCs, CD14^+^ DDCs, CD1a^+^ DDCs and LCs among CD45^+^HLA-DR^+^Lin^−^ viable cells isolated from human eyelid skin samples. Representative data from two independent experiments are shown.

APCs mediate cellular immune responses by processing and presenting antigens for the recognition by T cells. We next analyzed whether the M.CoIV_6 h protocol enables the isolation of major types of human skin APCs. The following five major described types of APCs in the human skin (24) were characterized, namely, (1) plasmacytoid dendritic cells (pDCs), (2) conventional dendritic cells (cDCs), (3) CD14^+^ dermal dendritic cells (CD14^+^ DDCs), (4) CD1a^+^ dermal dendritic cells (CD1a^+^ DDCs), and (5) Langerhans cells (LCs) (Figures 4A,B). Among *ex vivo* lineage negative human skin lymphocytes (CD45^+^HLA-DR^+^DUMP^−^), pDCs were rare while cDCs were relatively abundant (0.27 vs. 11.75%), which is consistent with previous findings (22, 23), that the low levels of CD303 expression by skin pDCs were not due to the downregulation that might be potentially induced by the isolation procedure (Supplementary Figures 5C,D). Additionally, CD1a^+^DDCs (37.91%), CD14^+^ DDCs (3.00%), and LCs (5.00%) could also be identified (Figure 4C). Thus, the M.CoIV_6 h protocol is capable of effectively isolating various types of APCs from human skin tissues.

### *Ex vivo* Skin T Cells Exhibit Functional Capacities

**Figure 5:**
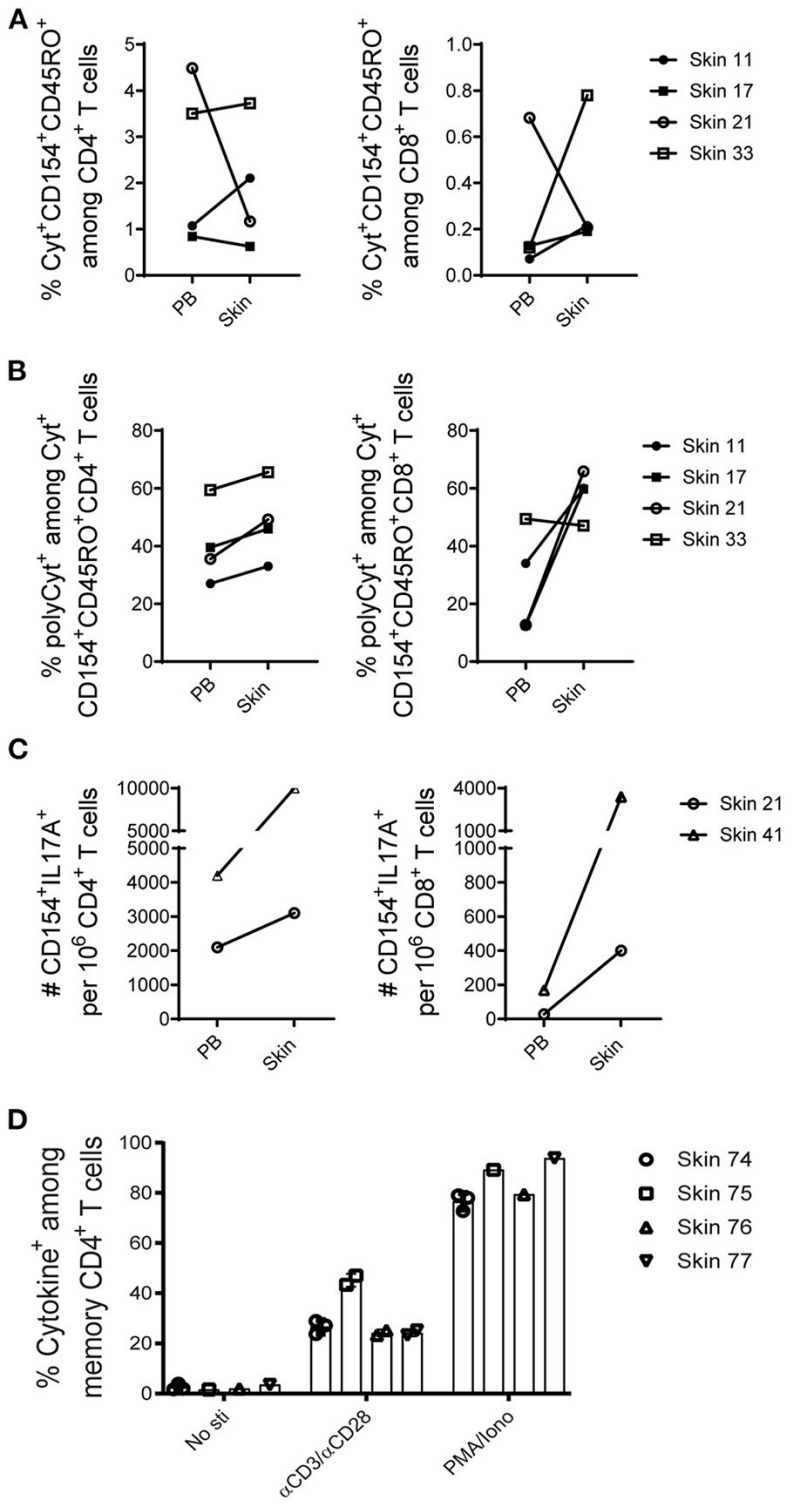
Functional capacities of T cells from skin and paired peripheral blood samples. **(A–C)**, mononuclear cells isolated from five paired skin and PB samples were stimulated with the SEB, and the induced cytokine production (IFN-γ, IL-2, or TNF-α; alternatively IFN-γ, IL-2, TNF-α, or IL-17A) in memory CD4^+^ and CD8^+^ T cells was examined according to CD154 expression. For each subpopulation, the background (as detected in the anti-CD28 stimulated but otherwise equally treated control samples) was subtracted. **(A)** Antigen-specific CD154^+^cytokine^+^ (total cytokine-producing) memory CD4^+^ and CD8^+^ T cells are shown in frequencies among CD4^+^ and CD8^+^ T cells. **(B)** The proportions of polyCyt^+^-producing (more than one of the analyzed three or four cytokines TNF-α, IFN-γ, IL-2, or IL-17A) memory T cells among cytokine^+^CD154^+^CD45RO^+^ CD4^+^ and CD8^+^ T cells. **(C)** The absolute numbers of IL-17A^+^ cells per million CD4^+^ and CD8^+^ T cells isolated from two analyzed paired skin and blood samples upon SEB stimulation are shown. **(D)** Percentage of cytokine^+^ cells among skin memory CD4^+^ T cells in response to αCD3/αCD28 stimulation. No antigens and PMA/Ionomycin stimulation were included as controls. Bars with two or three data points are shown as the mean of replicates of cells analyzed from each sample.

To validate whether memory T cells isolated from human skin are functional, cytokine profiles of cells upon *ex vivo* antigenic stimulation were evaluated in comparison with paired blood memory T cells (Supplementary Figure 6). Skin and blood mononuclear cells were stimulated with the super antigen SEB and CD28 antibodies for 7 h. Memory CD4^+^ T cells reacting to the antigen were identified according to the expression of CD154 (25, 26) and one or more of the cytokines TNF-α, IFN-γ, IL-2, or IL-17 as assessed by intracellular immunolfluorescence (7). T cells that have two or more functions, such as the production of cytokines, are polyfunctional. Polyfunctionality of T cells is associated with enhanced protection (27). In response to the stimulation with SEB, CD154^+^cytokine^+^ cells were readily detectable both in blood and skin with comparable frequencies (Figure 5A) and absolute cell numbers (data not shown). Among four matched samples, the fraction of polyfunctional cytokine-producing (polyCyt^+^) T cells were higher in memory CD4^+^ T cells from skin than blood (Figure 5B). Likewise, higher frequencies of polyCyt^+^ memory CD8^+^ T cells were found from skin than blood in three out of four donors (Figure 5B). In terms of the expression of IL-17A, both skin CD4^+^ and CD8^+^ T cells secreted more IL-17^+^CD154^+^ cells than their blood-derived counterparts in two analyzed donors (Figure 5C). In addition, on average about 30% of skin memory T cells responded to the stimulation with anti-CD3 and anti-CD28 (Figure 5D, Supplementary Figure 7).

**Figure 6:**
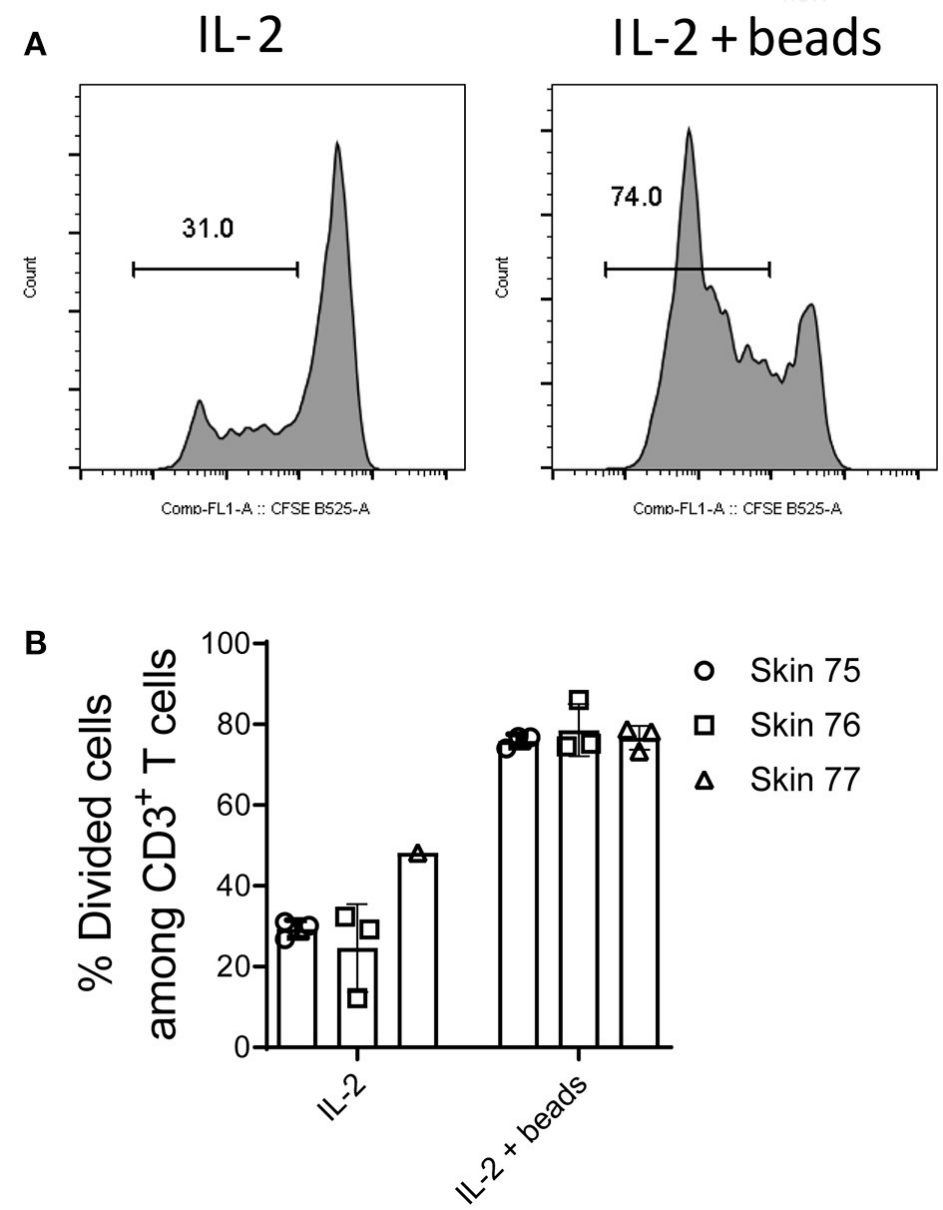
Long-term cultures and expansion of *ex vivo* isolated skin T cells. Skin mononuclear cells were isolated using the optimized M.CoIV_6 h isolation protocol from three individual skin samples. **(A)** Representative histograms and **(B)** percentages of divided cells among skin CD3^+^ T cells cultured in medium supplemented with Proleukin (IL-2) (left) or in the presence of additional T cell expansion beads (right). In **(B)** bars with three data points represent the mean (± SEM) of three replicates of cells analyzed from each sample.

Finally we evaluated whether memory T cells isolated from human skin could be used for antigen-specific responses and other parameters that may require longer periods of time in *ex vivo* cultures. To this end, skin cells were isolated using the optimized M.CoIV_6 h protocol and further examined after 5-day *ex vivo* cultures for their viability and proliferation potential. When cultured in medium alone, the number of skin T cells on day 5 remained similar to that of day 0 (data not shown). Of note, when cultured in medium supplemented with IL-2, about 30% of skin T cells had proliferated (Figure 6A) on day 5, that more than 70% of proliferation was observed in cultures in the presence of additional anti-CD3 and anti-CD28 (Figure 6B). Together, these results demonstrate that expanded skin (T) cells can be used for further downstream applications.

## Discussion

We report in this study optimization of rapid and efficient isolation protocols for characterizing human skin T_RM_ cells, in comparison with their matched blood counterparts. To date, human cutaneous αβ^+^ T cells *in situ* have been characterized mostly by immunohistochemistry staining (19, 28, 29), which might be biased either by the reaction itself or by incorrect interpretation (30). In the present report, by applying immunofluorescence staining techniques, we showed that CD4^+^ and CD8^+^ T cells can be detected both in the epidermis and dermis, with CD4^+^ T cells predominantly detected in the dermis (especially clustered around hair follicles). Our findings are supported by other studies (31–33) describing the T_RM_ cell tropism to the epidermis and follicles as epidermotropism. Studies in animal models showing that the preferential location of CD4^+^ and CD8^+^ T_RM_ cells in skin demonstrate their immediate local immune surveillance and protective responses at the site of antigen exposure (1, 2, 17, 34). The loose spatial structure of the dermis shaped by an abundant extracellular matrix may facilitate the interaction between CD4^+^ T helper cells with other immune cells and non-immune components, e.g., hair follicles (35). Studies in mice have demonstrated that after HSV infection, memory CD4^+^ T cells are recruited and formed clusters around hair follicles in a CCL5-dependent manner (32). Moreover, hair follicle keratinocyte-derived IL-15 has been described to be required for the maintenance of CD8^+^ T_RM_ cells, and IL-7 for CD8^+^ and CD4^+^ T_RM_ cells (31). Therefore, hair follicles may be a preferred site of pathogen exposure and thus, for locating T_RM_ cells. On the other hand, our histological data also showed a close proximity of CD3^+^ T cells to CD1a^+^ DCs, which may facilitate antigen presentation and provision of other survival signals by CD1a^+^ DCs to T_RM_ cells. Interestingly, only cells in the epidermis but not T cells express CCR7 and Ki-67, which is a feature of keratinocytes (36).

To study the human skin T_RM_ cells, several isolation methods were reported (20, 21). However, these methods either suffer from low yield or require long-term *in vitro* culture periods. To overcome these challenges, here we have established an optimized protocol for rapidly isolating skin T_RM_ cells by the combinatorial and sequential procedures of a short period of collagenase IV digestion and a gentle mechanical tissue dissociation. As the dermis has an abundant extracellular matrix comprised of collagen and elastin fibers (35), different types of collagenases [I (37), 1A (38), or IV (21)] alone or in combination have been applied to break down these extracellular structures. In particular, type IV collagenase has a lower tryptic activity and high collagenase activity, which limits the damage to membrane proteins and receptors while effectively breaking down the collagen-rich dermal tissues, resulting in the effective release of intact T_RM_ cells for downstream isolation. However, isolation of skin T cells with collagenase IV (19) or enzyme alone (20, 21) is not effective in isolating large number of T cells. Indeed, among the six analyzed protocols, only the M.CoIV_6 h enabled a high yield of viable total mononuclear cells and T cells, on average 2.8 × 10^5^ cells per cm^2^ of skin. Based on the reasonable estimate that the number of T cells in 1 cm^2^ of skin is 1.1 × 10^6^ (19), we were able to isolate more than 20% of proportioned skin T cells, which is comparable with skin T cells isolated using the skin explant cultures (19). Additionally, although we observed slightly lower frequencies of CCR7^+^ T cells from *ex vivo* than from *in situ*, it has been shown that neither collagenase digestion nor mechanical dissociation method modify the expressions of both CLA and chemokine receptors, such as the CCR4, CCR6, CCR8, and CCR10, on isolated *ex vivo* skin T cells (20). Thus, the M.CoIV_6h protocol should not alter features of skin T cells. In fact, the M.CoIV_6 h protocol also best preserved critical expressions of surface markers such as CD4, CD8, and CD69 on skin T cells. In line with their *in situ* status, isolated *ex vivo* skin T cells exhibit a memory phenotype, express the tissue-resident marker CD69 and the skin-homing receptor CLA but lack the expression of CCR7, Ki-67, and other putative activation markers, indicating their non-proliferating, inactive, and tissue resident status in the steady state.

In addition, using the M.CoIV_6 h protocol, not only memory T cells but also major types of APCs could be effectively isolated from fresh human skin tissues, which allowed for a further assessment of the functionalities of skin T cells. In line with our previous findings of preferential enrichment of polyfunctional memory T cells in the human bone marrow (7), polyfunctional memory T cells are also more frequent in the skin than blood. This observation suggests that there is a preferential location of memory T cells in the skin with distinct antigen exposure experience, such as to *Candida albicans* (39), as evidenced by their higher amount of IL-17 production. The *ex vivo* skin T cell responses are likely an attribute of the effective isolation of various types of APCs. Thus, this optimized protocol could help pave the way for research in human skin T_RM_ cells as such and, in particular, in direct comparison with their blood-circulating counterparts at the same sampling time.

## Materials and Methods

### Study Cohort

This study was approved by the ethics committee at the Charité – Universitätsmedizin Berlin, Germany (EA1/290/14). All blood and skin tissue samples were obtained with informed consent from all donors. Samples taken from normal adult skin with paired peripheral blood samples (mean age ± SEM, 67.29 ± 3.55 *y*; *n* = 14) or without (mean age ± SEM, 58.42 ± 2.90 *y*; *n* = 24) were obtained from healthy donors undergoing plastic cutaneous surgeries (Supplementary Table 1).

### Histological Staining

Skin samples were immediately fixed in 2% paraformaldehyde (Carl Roth) for 4 h at 4°C. Following fixation, samples were sequentially equilibrated in solutions supplemented with 10–30% sucrose (Carl Roth), each for 24 h at 4°C. Samples were then embedded in O.C.T^TM^ media (SAKURA) and stored at −80°C until cryosectioning using Kawamoto's tape method (40) with a microtome MH560 cryostat (Thermo Fisher). Tissue sections in 6 μm were blocked with blocking buffer (PBS with 0.1% Tween 20, and 10% FCS) for 1 h at room temperature and then stained with primary and secondary antibodies as well as DAPI (2 μg/mL) to label cell nuclei. Among the used anti-human antibodies, anti-CD3 Alexa Fluor 594 (UCHT1), anti-CD4 Alexa Fluor 555 (TT1), anti-CD8 Alexa Fluor 647 (GN11/134D7), anti-CD1a Cy5 (OKT6) were conjugated in house. Other antibodies include anti-CD69 Alexa Fluor 488 (FN50; Biolegend), anti-CCR7 Alexa Fluor 555 (Y59; Abcam), Anti- Ki-67 Biotin (SolA15; eBioscience), anti-CLA APC (HECA-452; Miltenyi) and streptavidin Alexa Fluor 488 (Thermofischer).

Following staining, the sections were mounted with fluorescent mounting medium (DAKO). Confocal images were generated using a Zeiss LSM710 (Carl Zeiss). Skin section picture composites were generated by three-dimensional tile scanning using a Plan-Apochromat 20X (0.8 numerical aperture; NA) air objective lens. The displayed overview image was part of 3 × 4 tile scans, with maximum intensity projections of z-stacks each with 1.3 μm z-resolution and x-y resolution of 7,578 × 5,734 pixels. Tiles were recorded with a 10% overlap and projections stitched together by the acquisition software to generate three high-resolution images. Images were analyzed using the ZEN software (blue edition).

For quantification of cells, the segmentation pipeline was designed using a previously described similar approach (41) and performed in Fiji, a distribution of ImageJ/Fiji (1.52p) (42). In every image set nuclei were identified by a plugin called “StarDist” (43). The objects were further used to measure the nuclear area and mean intensity in every staining. Signals above defined intensity thresholds were counted as positive signals. Counting of co-expressing cells was performed by using multiple thresholds for the markers of interest.

### Skin and Blood Sample Preparation

Peripheral blood mononuclear cells (PBMCs) were isolated by density gradient sedimentation using Ficoll-Paque^TM^ Plus (Sigma-Aldrich). Skin samples were delivered in CUSTODIOL® HTK solution (kindly provided by the Köhler Chemie, Germany) for <24 h until further preparation. In brief, skin samples were rinsed with cold PBS buffer, and the subcutaneous fat and hairs were carefully removed. Skin tissues were minced with sterile scissors into 2–4 mm fragments. About 25–50 fragments were digested in 3 mL digestion media in an incubator at 37°C and 5% CO_2_. Various components were used to digest skin fragments in different protocols (Supplementary Table 2), such as 0.8 mg/mL collagenase IV (Worthington), 0.4 mg/mL collagenase P (Roche), 1.25 mg/mL collagenase I (Sigma-Aldrich), 0.5 mg/mL elastase (Worthington), 0.5 mg/mL hyaluronidase (Worthington), 0.02 or 0.1 mg/mL DNAse I (Roche), 0.1 mg/mL trypsin inhibitor (Sigma-Aldrich) and 3.2 mm CaCl_2_·2H_2_O. RPMI1640 or DMEM culture medium (Thermo Fisher) was supplemented with 5% human AB serum (Sigma-Aldrich), 1% HEPES, 1% Pen/Strep (100 U/mL penicillin; 100 μg/mL streptomycin). The digestion procedure was terminated by adding an equal volume of PBS consisting of 2 mM EDTA. Skin fragments were then dissociated with a Gentle MACS Dissociator (Miltenyi Biotec). The homogenized tissue samples were further filtered through a 70 μm cell strainer (Miltenyi Biotec). If present, residual fragments were dissociated through a second dissociation step. Upon isolation, viable cells were quantified with DAPI using a MACSQuant. Digestion procedures using the whole skin dissociation kit with or without enzyme P (WSD+/-EnzP) (Miltenyi Biotec) were performed according to manufacturer's recommendation.

### *Ex vivo* Antigen Stimulation

Isolated mononuclear cells from the blood and skin were adjusted to a density of 1 × 10^7^ cells/mL in culture medium. Cells were stimulated with 1 μg/mL Staphylococcus Enterotoxin B (SEB) (Sigma-Aldrich), plate bound αCD3/αCD28 (Thermo Fischer; each 1 μg/mL) or PMA (1ng/mL) plus Ionomycin (1 μg/mL) (Thermo Fischer) for 7 h at 37°C, 5% CO_2_, with 5 μg/mL Brefeldin A (Biolegend) added during the last 2 h. Cultured cells without added antigen served as negative controls.

### Cell Surface and Intracellular Staining for Flow Cytometry Analysis

Up to 10 million cells were stained with antibodies and Fc Blocking reagent (Miltenyi Biotec) for 10 min in the dark at 4°C. When staining with the anti-CCR7 antibody, cells were stained for 15 min in the dark at 37°C. To detect the intracellular production of cytokines, stimulated cells were fixed with 2% paraformaldehyde followed by permeabilization (Perm 2; BD Biosciences), prior to intracellular CD154 and cytokine staining. The following fluorochrome-conjugated mouse anti-human antibodies were used to stain cells: anti-CD45 PE-vio770 (5B1), anti-CD45 APC-vio770 (5B1), anti-CLA APC (HECA-452), anti-CD25 APC (REA570), anti-CD11c Percp-vio770 (MJ4-27G12), anti-CD207 Pe-vio770 (MB22-9F5) and anti-CD1c FITC (AD5-8E7) (Miltenyi Biotec), anti-CD45 BV785 (HI30), anti-CD3 A700 (HIT3a), anti-CD8 BV785 (RPA-T8), anti-CD69 BV421 (FN50), anti-CD154 BV421 (24-31), anti-HLA-DR APC-Cy7 (L243), anti-CD45RO BV650 (UCHL1), anti-CCR7 A488 (G043H7), CD20 BV510 (2H7), CD34 BV510 (581), CD56 BV510 (HCD56), CD14 BV605 (M5E2), CD303 BV421 (201A), IL-2 FITC (MQ1-17H12), and IFN-γ PE-Cy7 (B27) (Biolegend), anti-CD3 APC-H7 (Sk7), anti-CD3 V500 (UCHT-1), anti-CD19 V500 (HIB19), CD141 BV711 (1A4) and TNF APC (MAb11) (BD Biosciences), anti-CD4 Pe-Cy5.5 (Sk3), anti-Ki-67 PE (20Raj1), anti-CD137 FITC (4B4) and IL-17 PE (eBio64DEC17) (eBioscience), and anti-CD14 Pacific Orange (TM1), anti-CD19 Pacific Orange (BU12) and CD1a Cy5 (OKT6) (house conjugate). Stained cells were acquired using a MACSQuant (Miltenyi Biotec) or a LSRFortessa (BD Biosciences) flow cytometer. At least 1 × 10^6^ lymphocytes were acquired. The data were analyzed with Flowjo V10 (Tree Star).

### CFSE Labeling and Long-Term Cell Culture

Freshly isolated skin mononuclear cells using the M.CoIV_6 h isolation protocol were labeled with Carboxyfluorescein succinimidyl ester (CFSE) at the final concentration of 2.5 μM. Briefly, cells were washed twice in PBS and the cell pellet was resuspended in PBS at density of 10 × 10^6^ cells/mL, and then labeled with 2.5 μM CFSE at 37°C for 10 min. The reaction was stopped by adding 5 mL FCS and washed twice. Labeled skin cells were cultured in X-vivo 15 medium (Lonza) containing 10% human AB serum and 500 IU/mL Proleukin (IL-2; Novartis) as well as 1% Pen/Strep for 5 days. Fractions of skin cells were additionally stimulated with T activation/expansion beads (Miltenyi Biotec) at a bead-to-cell ratio of 1:1.

### Statistics

Statistical analyses were performed with Graphpad Prism software (version 5.04). For analysis of two groups, two-tailed Wilcoxon matched-pairs signed rank test or unpaired *T*-test with Welch's correction was used, and a *p*-value under 0.05 was considered statistically significant.

## Data Availability Statement

The raw data supporting the conclusions of this article will be made available by the authors, without undue reservation.

## Ethics Statement

The studies involving human participants were reviewed and approved by the ethics committee at the Charité University Medicine, Berlin, Germany (EA1/290/14). The patients/participants provided their written informed consent to participate in this study.

## Author Contributions

JD: conceptualization, writing – review & editing, supervision, and project administration. WD, DL, RK, AEH, and JD: methodology. EZ, JW, and JB: sample resource. WD: investigation and writing – original draft. WD, RK, CC, JL, and MM: analysis. WD and JD interpretation. JD and AR funding acquisition. All authors contributed to the article and approved the submitted version.

## Conflict of Interest

The authors declare that the research was conducted in the absence of any commercial or financial relationships that could be construed as a potential conflict of interest.
