# Supplementary material for: Rapid Isolation of Functional ex vivo Human Skin Tissue-Resident Memory T Lymphocytes
Source: Front Immunol. 2021 Mar 22;12:624013. doi: 10.3389/fimmu.2021.624013 (PMC8019735; doi:10.3389/fimmu.2021.624013)
Supplement: Supplementary file 1 [file Data_Sheet_1.pdf]

Supplementary Material

1 Supplementary Figures and Tables

1.1 Supplementary Figures

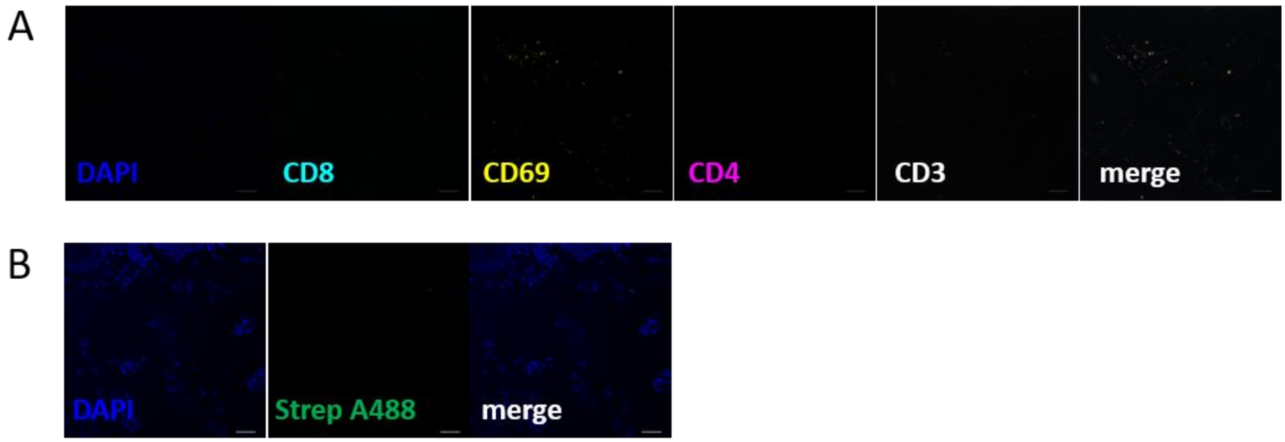

Supplementary Figure 1. Controls in histological staining. (A) Images of a skin section without primary antibody staining. (B) Images of a skin section with only secondary antibody streptavidin A488.

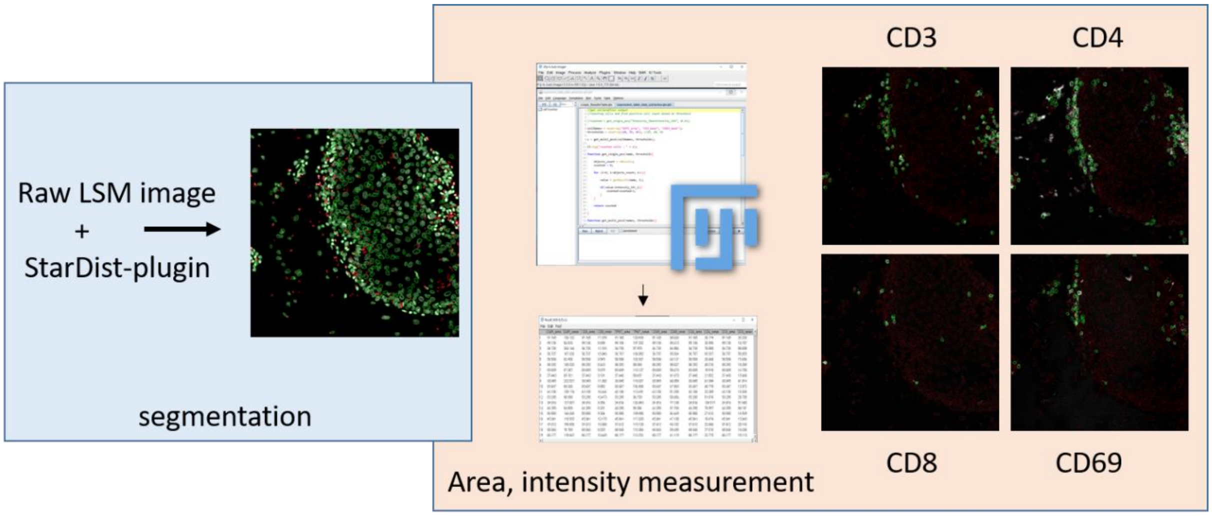

Supplementary Figure 2. Image based cell quantification in Fiji. The raw nuclei images (stained with DAPI) were used as input for the StarDist PlugIn, finding single cell objects. With help of the found objects their mean area and channel dependent mean intensity were measured. Cell objects expressing mean intensity above the user defined threshold were counted as positive cell. Counting of co-expressing cells was performed by defining multiple thresholds.

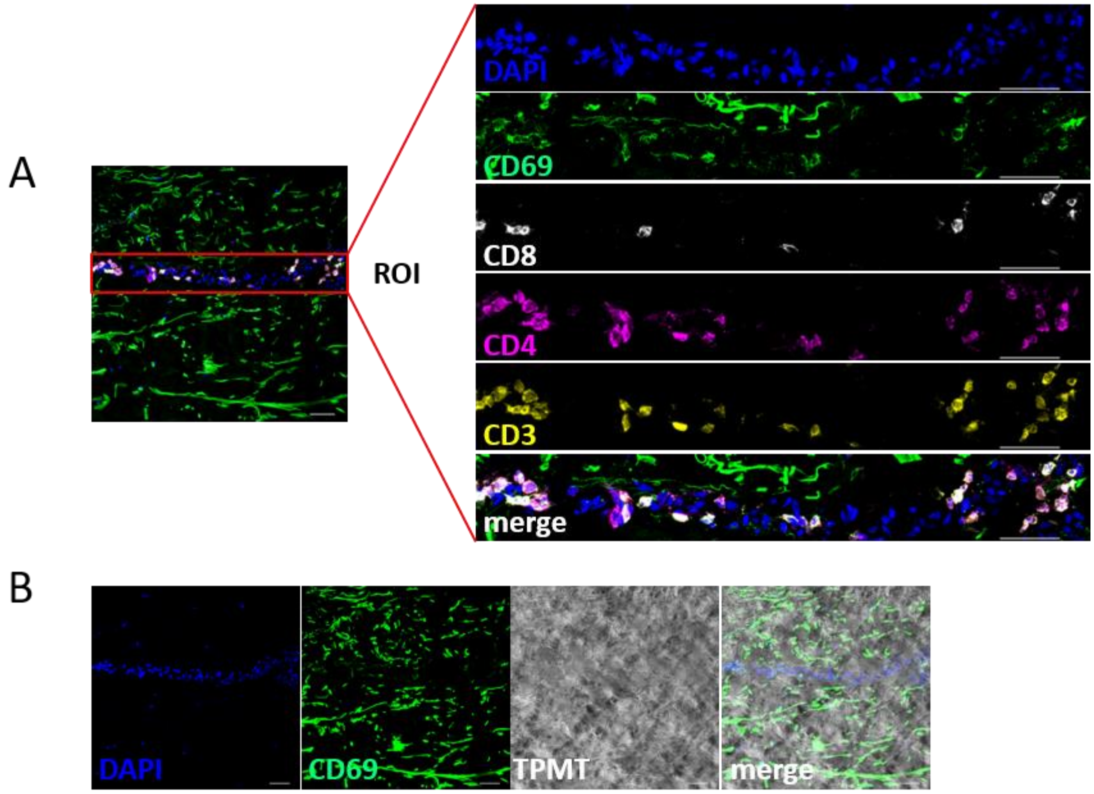

Supplementary Figure 3. T cells in normal human abdominal skin. (A) Images of an abdominal skin section stained with DAPI, CD69, CD8, CD4, CD3, or merged with all the markers. (B) Images of an abdominal skin section by CD69 staining overlap with transmitted light detector (TPMT).

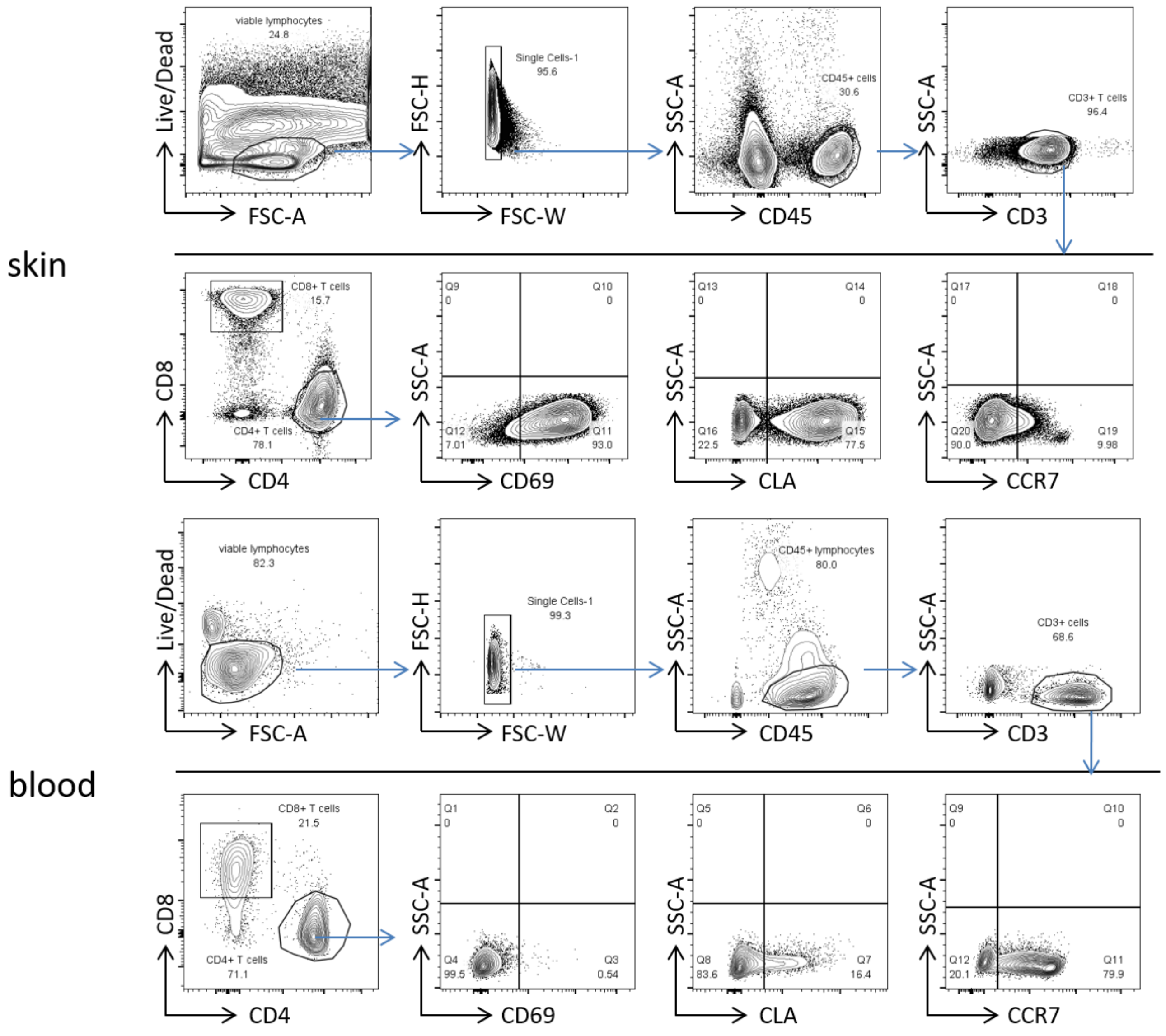

Supplementary Figure 4. Gating strategy for analyzing *ex vivo* skin and blood T cells. Viable lymphocytes were gated based on FSC-A against live/dead marker. Doublets were excluded and cells were then gated on $CD45^+$ and $CD3^+$ T cells. The expression levels of CD4, CD8, CD45RA, CD45RO, CD69, CLA, and CCR7 on T cells from skin and blood samples are shown. Data are representative of more than 10 independent experiments.

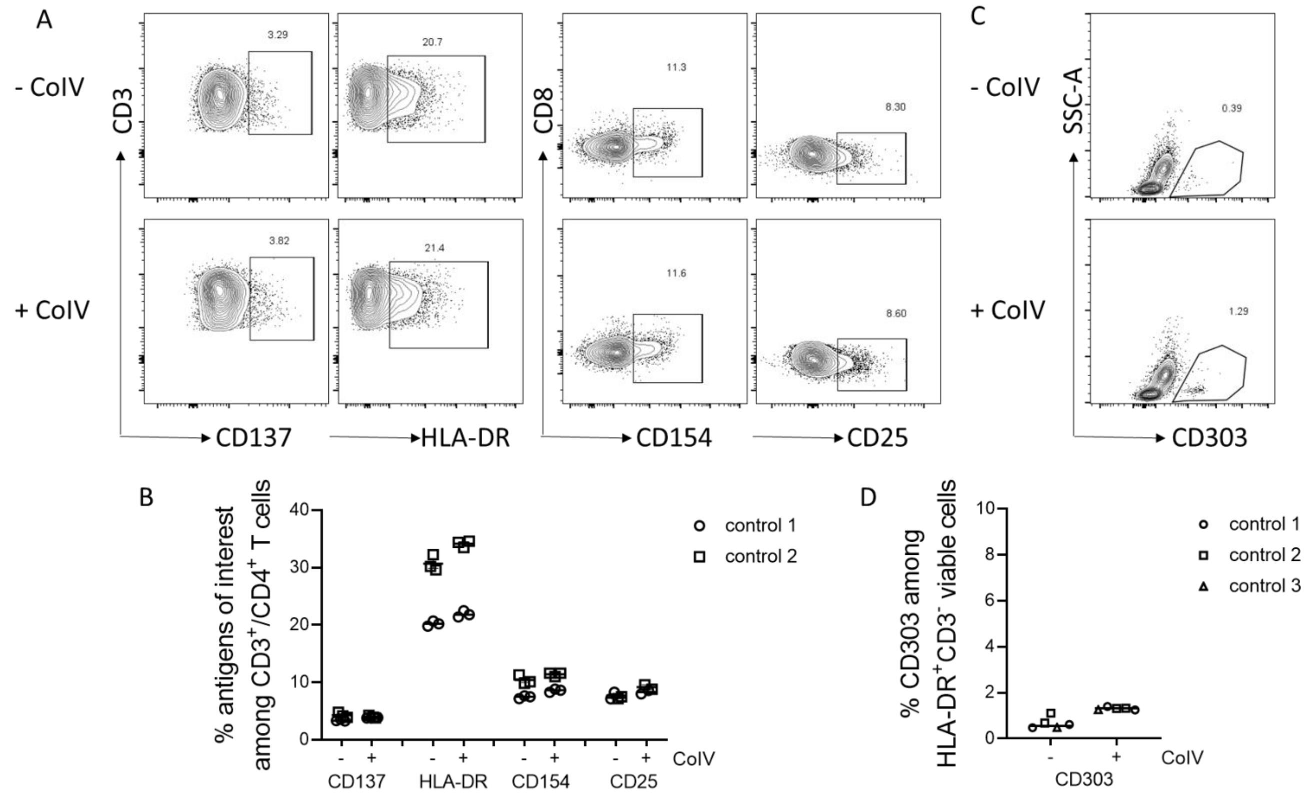

Supplementary Figure 5. Collagenase IV digestion has minimum effects on the expression of antigens of interest on PBMCs. PBMCs pre-activated with SEB for 12h (A, B) or directly ex vivo (C, D) were cultured in medium supplemented with (+ CoIV) or without (- CoIV) collagenase IV. Effects on the expression of activation markers by pre-activated cells are shown by representative plots (A) and by percentages among viable $CD3^+$ or $CD4^+$ T cells. Effects on the expression of CD303 on resting cells are shown by representative plots (C) and by percentages (D) among HLA-DR^+CD3^- viable cells.

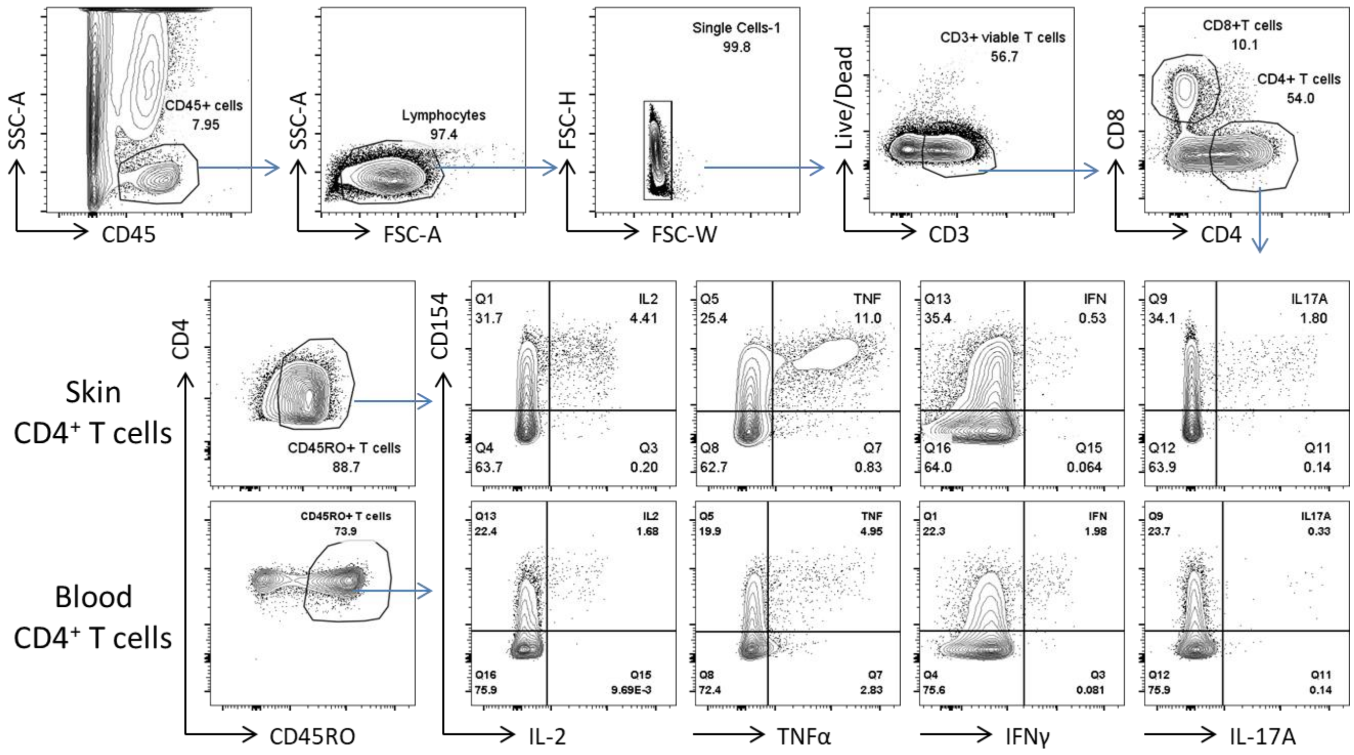

Supplementary Figure 6. Gating strategy for analyzing cytokine production by ex vivo skin and paired blood T cells upon antigen stimulation. Mononuclear cells isolated from blood as well as from skin cells by the M.CoIV_6h protocol were stimulated with SEB for 7 hours, with Brefeldin A added during the last 2 hours. Cells were then fixed and stained with T cell lineage and cytokine markers. Viable $CD3^+$ T cells were gated on $CD45^+$ single lymphocytes followed by $CD4^+$ and $CD8^+$ T cells (upper row). The expression levels of cytokines including IL-2, TNF-α, IFN-γ or IL-17A that co-express with CD154 on CD45RO memory $CD4^+$ from skin (middle row) and blood (lower row) are shown. Data are representative from 4 independent experiments.

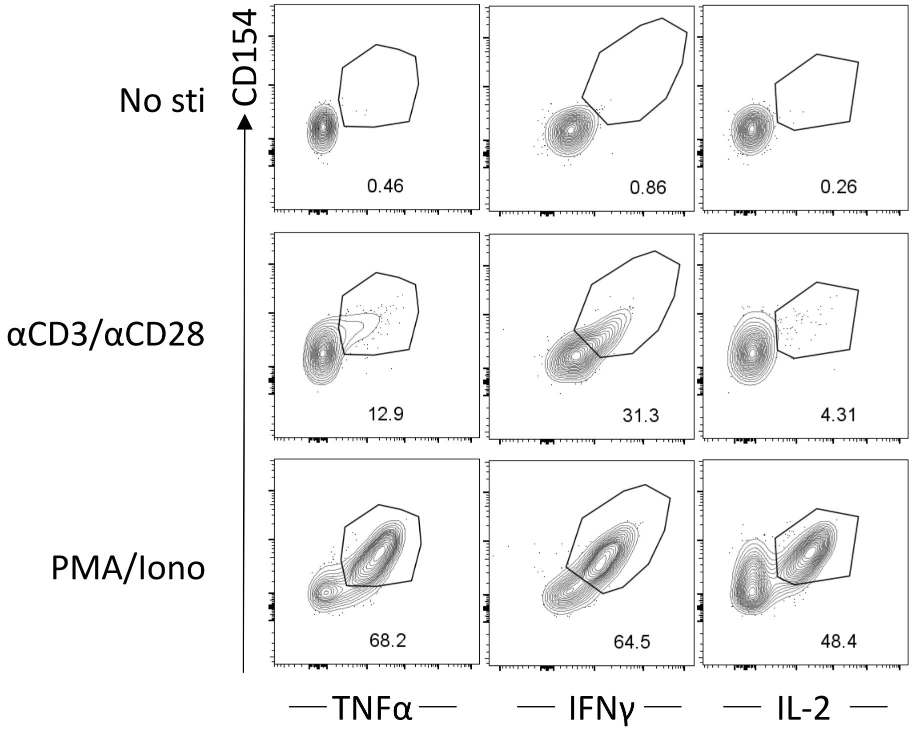

Supplementary Figure 7. Functional profiling of poly-reactive $CD4^+$ T cells from the human skin. Skin cells were isolated using the M.CoIV_6h method and cultured in medium alone, stimulated with plate-bound anti-CD3/CD28, or PMA/Iono for 7 hours, with Brefeldin A added during the last 2 hours. Cells were then analyzed for intracellular cytokines, TNF-α, IFN-γ, or IL-2. Contour plots of cytokines e.g. TNF-α, IFN-γ, or IL-2 that co-express with CD154 on memory $CD4^+$ skin T cells.

1.2 Supplementary Tables

Sample No.	Donor	Gender	Age (y)	Location	paired blood
1	Sk1	M	49	Eyelid	no
2	Sk2	F	66	Eyelid	no
3	Sk9	F	71	Eyelid	yes
4	Sk10	F	69	Eyelid	no
5	Sk11	F	83	Eyelid	yes
6	Sk12	F	83	Eyelid	no
7	Sk13	F	54	Eyelid	yes
8	Sk14	F	54	Eyelid	no
9	Sk17	M	74	Eyelid	yes
10	Sk18	F	60	Eyelid	yes
11	Sk19	F	60	Eyelid	no
12	Sk20	M	74	Eyelid	no
13	Sk21	n.a.	40	Inner leg	yes
14	Sk22	F	35	Abdomen	no
15	Sk33	F	57	Eyelid	yes
16	Sk34	F	63	Eyelid	no
17	Sk35	F	60	Eyelid	no
18	Sk36	F	82	Eyelid	yes
19	Sk40	F	63	Arm	yes
20	Sk41	F	54	Breast	no
21	Sk44	M	77	Eyelid	yes
22	Sk45	F	73	Eyelid	no
23	Sk56	M	67	Eyelid	no
24	Sk57	F	80	Eyelid	yes
25	Sk58	F	38	Abdomen	no
26	Sk59	F	51	Eyelid	yes
27	Sk60	M	79	Eyelid	yes
28	Sk61	F	25	Breast	no

29	Sk64	F	55	Abdomen	no
30	Sk66	F	57	Eyelid	no
31	Sk67	F	46	Abdomen	no
32	Sk71	F	71	Eyelid	no
33	Sk72	F	71	Eyelid	yes
34	Sk73	F	43	Breast	no
35	Sk74	F	81	Eyelid	no
36	Sk75	M	61	Eyelid	no
37	Sk76	F	63	Eyelid	no
38	Sk77	F	55	Eyelid	no

Supplementary Table 1. Donor information. Sample/donor ID, sex, age, skin sample location and blood pairing information are shown. F: female, M: male, n.a.: not available. (n = 38).

Protocols tested	Components	Digestion medium	Digestion time
M. CoIV_12h	0.8 mg/mL Collagenase IV, 0.02 mg/mL DNase I	RPMI1640	12 hours
M. CoIV_6h	0.8 mg/mL Collagenase IV, 0.02 mg/mL DNase I	RPMI1640	6 hours
WSD+EnzP_12h	50 µL Enzyme D, 2.5 µL Enzyme A, 12.5 µL Enzyme P	435 µL buffer L	12 hours
WSD-EnzP_12h	50 µL Enzyme D, 2.5 µL Enzyme A	435 µL buffer L	12 hours
CoP+IV_12h	0.4 mg/mL Collagenase P (w/o 0.4 mg/mL Collagenase IV), 0.02 mg/mL DNase I	RPMI1640	12 hours
Cocktail_3h	1.25 mg/mL Collagenase I, 0.5 mg/mL Elastase, 0.5 mg/mL Hyaluronidase, 0.1 mg/mL trypsin inhibitor, 3.2 mM $CaCl_2 \cdot 2H2O$, 0.1 mg/mL DNase I	DMEM	3 hours

Supplementary Table 2. Composition and enzymatic digestion time of different protocols. Protocols, components, digestion medium and time are listed.
